# Perioperative digital behaviour change interventions for reducing alcohol consumption, improving dietary intake, increasing physical activity and smoking cessation: a scoping review

**DOI:** 10.1186/s13741-021-00189-1

**Published:** 2021-07-06

**Authors:** Katarina Åsberg, Marcus Bendtsen

**Affiliations:** Department of Health, Medicine and Caring Sciences, Division of Society and Health, Linköping University, 581 83 Linköping, Sweden

**Keywords:** Perioperative, Digital behaviour change interventions, Lifestyle behaviour, Feasibility, Randomised controlled trial, Scoping review

## Abstract

**Background:**

Evidence suggests that unhealthy lifestyle behaviours are modifiable risk factors for postoperative complications. Digital behaviour change interventions (DBCIs), for instance text messaging programs and smartphone apps, have shown promise in achieving lifestyle behaviour change in a wide range of clinical populations, and it may therefore be possible to reduce postoperative complications by supporting behaviour change perioperatively using digital interventions. This scoping review was conducted in order to identify existing research done in the area of perioperative DBCIs for reducing alcohol consumption, improving dietary intake, increasing physical activity and smoking cessation.

**Main text:**

This scoping review included eleven studies covering a range of surgeries: bariatric, orthopaedic, cancer, transplantation and elective surgery. The studies were both randomised controlled trials and feasibility studies and investigated a diverse set of interventions: one game, three smartphone apps, one web-based program and five text message interventions. Feasibility studies reported user acceptability and satisfaction with the behaviour change support. Engagement data showed participation rates ranged from 40 to 90%, with more participants being actively engaged early in the intervention period. In conclusion, the only full-scale randomised controlled trial (RCT), text messaging ahead of bariatric surgery did not reveal any benefits with respect to adherence to preoperative exercise advice when compared to a control group. Two of the pilot studies, one text message intervention, one game, indicated change in a positive direction with respect to alcohol and tobacco outcomes, but between group comparisons were not done due to small sample sizes. The third pilot-study, a smartphone app, found between group changes for physical activity and alcohol, but not with respect to smoking cessation outcomes.

**Conclusion:**

This review found high participant satisfaction, but shows recruitment and timing-delivery issues, as well as low retention to interventions post-surgery. Small sample sizes and the use of a variety of feasibility outcome measures prevent the synthesis of results and makes generalisation difficult. Future research should focus on defining standardised outcome measures, enhancing patient engagement and improving adherence to behaviour change prior to scheduled surgery.

**Supplementary Information:**

The online version contains supplementary material available at 10.1186/s13741-021-00189-1.

## Introduction

Patients undergoing surgery are at risk of postoperative complications, which may result in increased postoperative morbidity and mortality, extended hospital stay and increased societal costs (Wakeam et al., 2015; Tevis et al., 2016). While the surgical procedure itself may be an unavoidable risk factor, evidence suggests that unhealthy lifestyle behaviours, such as alcohol, diet, physical activity and smoking are modifiable risk factors for postoperative complications (Thomsen et al., 2009; Eliasen et al., 2013; Steffens et al., 2018; Schwegler et al., 2010; Levett et al., 2016; Moller et al., 2003). Digital behaviour change interventions (DBCIs) are interventions that employ computer technology, usually websites, mobile phones or smartphone applications (apps), to encourage and support behaviour change with the goal of improving or maintaining health. DBCIs may be automated without the need for health professional input, but can be used in combination with digital and face-to-face support (Yardley et al., 2016). DBCIs have shown promise in achieving lifestyle behaviour change in a wide range of populations (Dorri et al., 2020; Villinger et al., 2019; Santo et al., 2018; Suffoletto et al., 2014; Hall et al., 2015; Müssener et al., 2016; Bendtsen et al., 2015; McCambridge et al., 2013; Bendtsen et al., 2012; Thomas et al., 2018; Balk-Moller et al., 2017; Buckingham et al., 2019), and it may therefore be possible to reduce postoperative complications by supporting behaviour change using digital interventions.

Perioperative medicine has been defined by Grocott and Mythen, 2015 (Grocott & Mythen, 2015) as “a patient-focused, multidisciplinary, and integrated approach to delivering the best possible health care throughout the perioperative journey from the moment of contemplation of surgery until full recovery”. Much of the perioperative medicine occurs outside the operating room such as behaviour change, prehabilitation and management of long-term health problems (Miller et al., 2016). In recent years, studies focusing on how to improve patients’ lifestyle behaviour preoperatively, with a view to improve postoperative recovery, have become more common (Gillis et al., 2018; Santa Mina et al., 2014; Wong et al., 2012; Mills et al., 2011; Martindale et al., 2013). For instance, a systematic review (Thomsen et al., 2014) concluded that preoperative smoking interventions may support short-term smoking cessation, which is important as smokers have been shown to be at a higher risk of respiratory complications during anaesthesia, as well as having increased risk of cardiopulmonary and wound-related postoperative complications (Thomsen et al., 2009; Moller & Tonnesen, 2006). Similarly, excessive preoperative alcohol consumption may increase the risk of postoperative morbidity, infections and wound and pulmonary complications (Eliasen et al., 2013). Intensive alcohol and smoking cessation interventions 6–8 weeks before surgery have been shown to reduce postoperative morbidity (Thomsen et al., 2014; Moller et al., 2002; Oppedal et al., 2012), which supports the causal argument. In addition, a recent systematic review found that intensive alcohol abstinence interventions may reduce the incidence of postoperative complications (Egholm et al., 2018).

Smoking and alcohol consumption are not the only lifestyle-related risk factors of postoperative complications. Low levels of physical activity may also increase the risk, and preoperative exercise has been associated with a reduced number of cases of postoperative pulmonary complications in lung cancer (Steffens et al., 2018) and cardiac patients (Snowdon et al., 2014; Marmelo et al., 2018). A systematic review concluded that preoperative physical therapy may reduce postoperative pulmonary complications and length of hospital stay in patients undergoing cardiac surgery (Hulzebos et al., 2012). There was also evidence that malnourished patients have a significantly higher postoperative mortality and morbidity (Schwegler et al., 2010; Goiburu et al., 2006; Wischmeyer et al., 2018).

Digital behaviour change interventions which support lifestyle behaviour change have shown promise in a range of clinical populations, for example among cancer and stroke patients (Zhang et al., 2018; Choi & Paik, 2018), and among others (Brewer et al., 2015; Burkow et al., 2018). While studies of lifestyle behaviour interventions in the surgical context are fewer, the research field of perioperative digital lifestyle interventions is growing, and it is therefore timely to take stock on the current state of the field.

### Objectives

As the body of literature on perioperative digital lifestyle behaviour change interventions has not been comprehensively reviewed, and since the topic is heterogenous and not amenable to more precise systematic review, we conducted a scoping review (Peters et al., 2015) in order to identify existing research done in the area of perioperative DBCIs for reducing alcohol consumption, improving diet, increasing physical activity and smoking cessation. This scoping review aims to describe the existing studies by population, intervention, comparator, outcomes and study design (PICOS), and findings may determine the value of a full systematic review (Munn et al., 2018).

## Methods

This review includes the items recommended by the PRISMA extension for Scoping reviews (Moher et al., 2009). A search strategy and plan for data extraction and synthesis was produced in advance but no protocol was published.

### Eligibility criteria

Eligibility criteria were developed using the PICOS format. Reports in peer-reviewed journals of quantitative, qualitative and mixed method studies were eligible for inclusion. Studies were included if they were published in English, without any restriction on publication date.

#### Participants

Studies including patients from any surgical specialty were included, with no restriction on type of surgery, participant gender or age. As there is no standardised definition of the duration of the perioperative period (Thompson et al., 2016), and in order to achieve a broad scope, this review covers all phases of perioperative medicine—from decision to perform surgery (e.g. referral or pre-assessment) until early postoperative inpatient hospital recovery period.

#### Interventions

Included interventions consisted of perioperative DBCIs. That is, interventions which employ computer technology, usually websites, mobile phones or smartphone applications (apps) to encourage and support behaviour change. The content of included interventions focused on supporting behaviour change with respect to at least one of: alcohol consumption, diet, physical activity or smoking. Interventions included were in principle unguided, and components of the interventions were delivered directly to participants via a digital device. Studies where an intervention was used only to schedule or remind participants of other activities (e.g. to take medicines) were not included, neither were disease- or recovery management systems such as physiotherapy or at home monitoring.

#### Outcomes

Studies were included if either behaviour change outcomes were reported (alcohol, diet, physical activity or smoking) or usability measures (including engagement, accessibility and user ratings).

### Information sources and search

We searched for literature in PubMed, PubMed Central; Cochrane Central Register of Controlled Trials (CENTRAL); Database of Abstracts of Reviews of Effects (DARE); Scopus; PsycINFO; PsycARTICLES; and Web of Science. A search strategy was created for PubMed, which was then adapted to the other information sources. The final search strategy for PubMed can be found in Additional file 1: Appendix A.

### Selection of sources of evidence and data charting process

The search results were exported into Mendeley and duplicates were removed. KÅ initially screened titles and abstracts of the identified articles and removed those clearly deemed irrelevant for the objective according to the predefined eligibility criteria. KÅ and MB thereafter independently screened the full texts of the identified articles and together reached a final decision on which studies to include. Data were extracted from the included studies by KÅ using the TIDieR checklist (Template for Intervention Description and Replication) (Hoffmann et al., 2014) and an author created form for extracting characteristics of studies (including PICOS, follow-up rates, results and funding). Table 1 shows the extracted data items. Finally, MB independently screened the data extraction for accuracy. Both KÅ and MB synthesized results. Qualitative findings were extracted as reported, and no attempt to further analyse findings were made.

**Table 1:** Items from the TIDieR checklist extracted from reports, and items extracted from studies via the study-specific form

**Items from the TIDieR checklist extracted from reports**	
1.	*Brief name* (item 1)
2.	*Why?* Rationale, theory or BCTs (item 2)
3.	*What?* Materials and procedures (item 3 + 4)
4.	*How was it provided?* Modes of delivery incl. ev. tailoring (item 6 + 9)
5.	*When and how much?* Timing in relation to surgery and “dose” (item 8)
6.	*Comparison group* (if any)
**Items extracted from studies via the study-specific form**	
7.	*Study design:* Study design, intervention (digital format, lifestyle behaviour) and type of surgery
8.	*Participants:* Patient group, sample size, gender and mean age of participants
9.	*Follow up:* Time of follow up and follow up rate
10.	*Outcomes:* Outcome measures, both quantitative and qualitative
11.	*Results:* Findings, both quantitative and qualitative
12.	*Funding:* The source of financial support

### Synthesis of results

The following sections were defined to aid the synthesis of results: populations and interventions, theoretical frameworks, feasibility studies, engagement data, behaviour change outcomes and protocols. KÅ synthesized the extracted data under each section in a descriptive overview. MB critically screened the process and synthesis, and findings were discussed in multiple sessions involving both KÅ and MB.

## Results

### Selection of sources of evidence

**Fig. 1:**
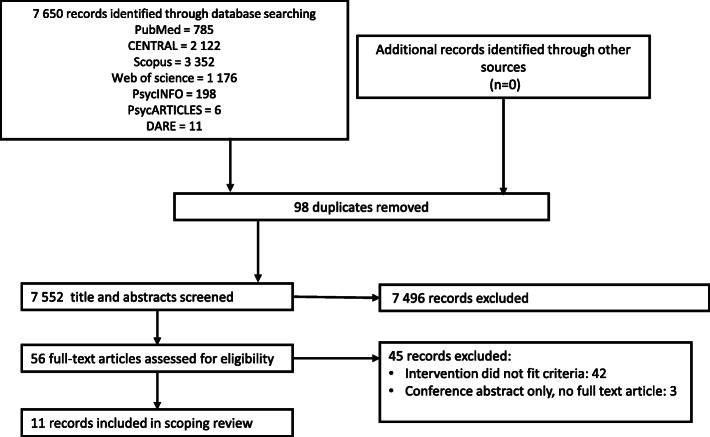
PRISMA flow diagram of record selection process

The search for records was conducted at three different dates in 2019: September 25 (PubMed), October 17 (CENTRAL, Scopus, Web of Science) and November 7 (PsycINFO, PsycARTICLES, DARE NHS-EED). On February 16, 2021, the search for records was repeated. Figure 1 shows a PRISMA flow diagram of the record selection process. Eleven records fulfilled the eligibility criteria and were subsequently included in the review.

### Characteristics and results of individual sources of evidence

A summary of included studies and their characteristics can be found in Table 2. Intervention descriptions including relevant items from the TIDieR checklist can be found in Additional file 2: Appendix B. Of the eleven studies included in this review, five (Lemanu et al., 2018; DeMartini et al., 2018; Krebs et al., 2019; van der Velde et al., 2021; Bendtsen et al., 2019) reported on randomised controlled trials (RCT) (full-scale trials = 1, pilot trials = 3, and study protocols = 1), and six (Mundi et al., 2015; McCrabb et al., 2017; Nolan et al., 2019; Low et al., 2020; Kulinski & Smith, 2020; Thomas et al., 2020) reported on feasibility studies. Most studies were conducted in the USA (*n* = 5), and the remaining in Australia, New Zealand, Netherlands and Sweden. The mean age of participants was 51.1 years (SD 9.0 years) (*n* = 385), and 57% were female (*n* = 413).

**Table 2:** Summary of included studies evaluating perioperative digital lifestyle interventions

Author, year (country) Study design Intervention Type of surgery	Participants (sample size) Gender Mean age (SD)	Follow up (rate)	Outcomes	Results	Strengths and limitations	Funding
Mundi et al., 2015 (United States of America) Feasibility Smartphone app (diet and physical activity) Bariatric (laparoscopic sleeve gastrectomy)	Bariatric surgery patients (*n* = 30) 27 women and 3 men 41.3 (11.4)	12 weeks (66.7%)	1. Bariatric surgery knowledge questionnaire (knowledge in areas of nutrition, physical activity and bariatric surgery) 2. International Physical Activity Questionnaire short form (IPAQ-SF) 3. Electronic survey (app user experience)	1. Small increase in nutrition knowledge. (26.0 ± 2.5 to 27.3 ± 2.0, *p* = 0.07) 2. Increase in self-reported minutes of vigorous activity. (25.5 ± 43.9 to 49.4 ± 51.1, *p* = 0.04) 3. Overall satisfaction with the app saying it helped them understand behaviours that may increase the likelihood of long-term weight loss (*n* = 19/20)	Strength: Approximately 70% of all modules were completed by users Limitation: Pre-post measures. Small sample size	Not disclosed
McCrabb et al., 2017 (Australia) Feasibility (pilot) Web-based smoking cessation program "Smoke-Free Recovery" (SFR) Orthopaedic (trauma)	Orthopaedic trauma patients (*n* = 31) Among those responding to follow-up (20): 6 women and 14 men 47.9 (14.3)	1–8 weeks post-discharge (64.5%)	1. Engagement data from the online platform 2. Semi-structured telephone interviews examining themes related to feasibility of the program (engagement, acceptability, and retention) and the smoking cessation process	1. A majority of participants (28/31) used the program during hospitalisation. Many responders (15/20) did not rate the program due to not remembering or technical issues during hospitalisation. One of the reasons for not remembering the program was medication related. Few responders (2/20) accessed the program after hospital discharge 2. Themes on program use included: Lack of time or need for additional support, computer illiteracy or technology issues, feeling unready or too stressed to quit, or feeling they had reached the boundary of what could be learnt from the program were all issues that affected program use	Strength: Detailed intervention description Limitation: Intervention delivery timing during hospitalisation	The National Health and Medical Research Council
Lemanu et al., 2018 (New Zealand) RCT Text messages (physical activity) Bariatric (laparoscopic sleeve gastrectomy)	Bariatric surgery patients (*n* = 102) 61 women and 27 men 43.8 (7.9)	Post-intervention (approx. 4–6 weeks) (86.3%) Post-operative (6 weeks) (73.5%)	Adherence to preoperative exercise advice (number of participants partaking in ≥ 450 metabolic equivalent minutes (METmin−1) exercise activity per week preoperatively) International Physical Activity Questionnaire (IPAQ)	Post-intervention: Adherence to exercise advice was significantly higher in exposure group (EG) than control group (CG) (*p* = 0.041). Median number of days responders partook in exercise activity was also significantly higher in EG than CG (*p* = 0.046). There was no difference in median weekly metabolic equivalent minutes (METmin−1) 6 week post-operative follow-up: There was no difference between the two groups for any outcome	Strength: RCT, standardised outcome measures and hypotheses Limitation: Underpowered, short intervention time to achieve improved physiological fitness in morbidly obese patients	Clinical Research Training Fellowship awarded by the Health Research Council of New Zealand
DeMartini et al., 2018 (United States of America) Pilot-RCT Text messages (alcohol) Transplantation (liver)	Transplant patients with alcoholic liver disease (ALD) (*n* = 15) 4 women and 11 men 50.80 (7.9)	8 weeks (93.3%)	1. Feasibility outcomes (participants’ intervention satisfaction ratings, 6/8 participants responded) 2. Efficacy outcomes (urine ethyl glucuronide (EtG) and self-reported alcohol measures)	1. Responders were satisfied with the intervention and found messages helpful for abstinence, coping with cravings and stress 2. In the intervention group, none of the individuals tested positive for biologically confirmed, objective alcohol consumption (e.g. ethyl glucuronide [EtG]) results at 8 weeks, however 2 out of 6 participants (33%) in the standard care group did test positive	Strength: Detailed intervention description, intervention results and participant satisfaction Limitation: Small sample size, convenience sample	Internal grant from the Psychological Medicine Service of Yale-New Haven Hospital
Bendtsen et al., 2019 (Sweden) RCT-protocol Text messages (smoking cessation) Elective	434* * Expected number of participants, recruitment not completed	3 months 6 months 12 months (N/A)	The following outcome measures will be used: Smoking cessation outcomes, prolonged abstinence, point prevalence of smoking abstinence, mediating factors			Kamprad Family Foundation for Entrepreneurship, Research & Charity
Nolan et al., 2019 (United States of America) Feasibility Text messages (smoking cessation) Elective	Elective surgery patients (*n* = 100) 47 women and 53 men 52.1 (9.7)	30 days postsurgery (95%)	1. Telephone survey (smoking behaviour, use of nicotine replacement therapy, feedback about content and frequency of messages, interest in using the text message service in the future) 2. Engagement data were recorded (average number of messages sent, and received per participant) 3. The program collected data on smoking status via text messages	1. High overall satisfaction (78/95) being somewhat or very satisfied on a 5 point Likert scale. (65/95) expressed that they would be somewhat or very interested in utilizing the text messaging service again for future surgeries. 25 participants (31%) reported 7-day point prevalence abstinence at 30-day telephone follow-up 2. Median number of text messages sent was 81, and median number of responses to prompts was 10 3. Selfreported 24-h abstinence rates ranged from 37% on day 2 to 71% on day 14	Strength: Intervention description, example messages, participant satisfaction Limitation: Brief reporting, pre-post measures	Internally by the Mayo Clinic
Krebs et al., 2019 (United States of America) Pilot-RCT Coping skills game for smoking cessation (Quit-IT) Cancer surgery (lung or gastrointestinal)	Cancer patients scheduled for surgical treatment (*n* = 39) 27 women and 11 men 57.4 (10.2)	1 month (61.5%)	1. Follow-up survey (phone or e-mail) 2. Satisfaction survey (captured in game) 3. Gameplay parameters (captured in-game) 4. Confirmed abstinence (biochemically verified)	1. A nonsignificant trend for increased confidence to quit smoking (situational self-efficacy) and higher intention to stay smoke free was found in the intervention group (*d* = 0.25, 95% CI −0.56 to 1.06) 2. 5/8 participants thought playing the game helped them cope with urges to smoke 3. Totally 8/20 in the intervention group played the game. Users completed an average of 2.5 episodes (range 1–10) Confirmed abstinence was higher in the intervention group (4/13) in relation to control group (2/11)	Strength: Theoretical and BCT underpinning intervention, detailed intervention description Limitation: Small number of users (*n* = 8/20) , underpowered sample size	National Institute of Health
Low et al., 2020 (United States of America) Feasibility (pilot) Smartphone app (physical activity/sedentary behaviour) Elective	Abdominal cancer surgery patients (*n* = 15) 12 women and 3 men 49.7 (11.5)	Daily assessed until 30 days postdischarge Weekly interviews (N/A)	Usability: (1) weekly ratings on a scale of 0 to 100 on easy of use; appearance, design, usability and intervention satisfaction and (2) via the System Usability Scale (ten-item questionnaire) at the end of intervention Feasibility: (1) accrual and retention rates, compliance with reporting symptoms. (2) Objective activity and heart rate data indicated compliance with wearing the smartwatch as well as walking in response to activity prompts. (3) Weekly semistructured interviews on usability, acceptability, and experience using the app	1. Usability. Participants rated the apps as very easy and pleasant to use. Overall satisfaction with the whole system was 89.9, and the mean System Usability Scale score was 83.8 out of 100 2. Feasibility. Compliance with the intervention declined significantly after surgery (and did not improve after discharge) for both symptom reporting and Fitbit. Fitbit compliance declined from before surgery (91%) to inpatient (36%) and postdischarge (65%) Participants generally reported that they liked the simplicity of the intervention and found the prompts to be motivating. Participants also reported that it was especially difficult to walk in the hospital immediately after surgery when they were too weak to walk unassisted, were in the middle of tests or other care procedures, or were on medications that made it difficult to get up and walk	Strength: Standardised outcome measures, objectively measured, detailed intervention description Limitation: Poor reporting on qualitative data, no behaviour change supportive content except "registration"	The UPMC Aging Institute and the National Cancer Institute
Kulinski and Smith, 2020 (Australia) Feasibility (pilot) Text messages (diet, physical activity, psychology and medical management) Elective	Patients with obesity undergoing (elective) surgery (*n* = 22) 9 women and 9 men Mean and sd not available, age range 24–77 years	6 months (81.8%)	Questionnaires (self-report, via telephone): 1. Lifestyle behaviour (weight, smoking, diet, exercise) (questionnaire not disclosed) 2. The generic health quality of life measure EuroQol-5 dimension 3-level questionnaire) (EQ-5D-3L) 3. Health engagement (Readiness to change scale, Rollnick, 1992) 4. User experience (enjoyment, recommendation to other people) and feasibility (recruitment and retention rates)	1. Self-reported improvements in lifestyle behaviour (weight, smoking, exercise). Self-reported dietary composition did not change 2. Eleven participants (61%) reported that their overall ‘health score’ improved after completing the programme 3. Seven participants (39%) rated their motivation to change their health as improved following involvement in the study 4. 15 of 18 participants (83%) found the programme useful or extremely useful. All stated that they would recommend the programme to others in the lead-up to elective surgery	Strength: Multiple lifestyle (physical activity and diet) Limitation: Pre-post measures, no standardised behavioural outcome measures, poor reporting of qualitative data	Department of Anaesthesia Research Fund, Wollongong Hospital, Wollongong, New South Wales, Australia
Thomas et al., 2018 (Sweden) Qualitative study Text messages (smoking cessation) Elective	Individual interviews with patients (*n* = 10) 4 women and 6 men Mean and sd not available, age range 45-70 years	3 months after the intervention (N/A)	Individual interviews: Semistructured format, included questions that aimed to capture patients’ experiences and use of the intervention	Patients showed strong motivation to quit smoking and an openness to incorporate the intervention into their behaviour change journey; however, the timing of the intervention and messages were important to optimize support. A text messaging, smoking cessation intervention can be a valuable and feasible way to reach smoking patients having elective surgery	Strength: Participants recruited among patients who visited a health care unit rather than out of convenience Limitation: No measures to ensure heterogeneity among participants	Kamprad Family Foundation for Entrepreneurship, Research & Charity
van der Velde et al., 2021 (Netherlands) Pilot-RCT Smartphone app (smoking, alcohol, physical activity, unintentional weight-loss) Major elective surgery	Patients with major elective surgery (*n* = 86) 39 women and 40 men 60.0 (5.2)	Pre-surgery (3 days prior to surgery) (58.1%) Post-surgery (30 days post-discharge) (73.2%)	Online questionnaires: 1. Usability (System Usability Scale) (3 days prior to surgery) 2. Change in risk behaviours (3 days prior to surgery) (questionnaire not disclosed) 3. Functional recovery (30 days post-discharge) (PROMIS-PF, Patient-Reported Outcomes Measurement Information System physical functioning 8-item short form) 4. Semi-structured telephone interviews (usability of the app) with 12 participants in the intervention group (pre- and postoperatively)	1. Patients considered the app to have acceptable usability (mean 68.2) [SD 18.4]) 2. Compared with the control group, the intervention group showed an increase in self-reported physical activity and muscle strengthening activities prior to surgery. Also, 2 of 2 frequent alcohol users in the intervention group versus 1 of 9 in the control group drank less alcohol prior to surgery. No difference was found in change of smoking cessation. 3. Between-group analysis showed no meaningful differences in functional recovery after correction for baseline values (β = – 2.4 [95% CI – 5.9 to 1.1]) 4. Interviews supported the usability of the app. The major point of improvement identified was further personalization of the app	Strength: Multiple lifestyle (physical activity, smoking, alcohol), detailed intervention description Limitation: Underpowered, lack of description of behaviour change outcomes	Foundation Innovative Alliance—Regional Attention and Action for Knowledge circulation

### Synthesis of results

#### Populations and interventions

The included studies covered a range of surgery types: bariatric (Lemanu et al., 2018; Mundi et al., 2015), orthopaedic (McCrabb et al., 2017), cancer (Krebs et al., 2019), transplantation (DeMartini et al., 2018) and non-specific elective surgery (van der Velde et al., 2021; Bendtsen et al., 2019; Nolan et al., 2019; Low et al., 2020; Kulinski & Smith, 2020). In the majority of studies, interventions were presented to patients prior to surgery, for instance at the preoperative planning meeting (Lemanu et al., 2018; DeMartini et al., 2018; van der Velde et al., 2021; Bendtsen et al., 2019; Mundi et al., 2015; Nolan et al., 2019; Low et al., 2020; Kulinski & Smith, 2020), and in two studies the interventions were introduced to patients in the ward post-surgery (Krebs et al., 2019; McCrabb et al., 2017). The majority of the interventions targeted smoking cessation (Krebs et al., 2019; Bendtsen et al., 2019; McCrabb et al., 2017; Nolan et al., 2019), physical activity (Lemanu et al., 2018; Low et al., 2020), or a combination of physical activity and diet (Mundi et al., 2015; Kulinski & Smith, 2020). Only two studies targeted either alcohol alone (DeMartini et al., 2018), or all four lifestyle behaviours at once (alcohol, diet, physical activity and smoking) (van der Velde et al., 2021). Studies varied in format: one game (Krebs et al., 2019), one web-based program (McCrabb et al., 2017), three smartphone apps (van der Velde et al., 2021; Mundi et al., 2015; Low et al., 2020) and five text message interventions (Lemanu et al., 2018; DeMartini et al., 2018; Bendtsen et al., 2019; Nolan et al., 2019; Kulinski & Smith, 2020). The text message interventions consisted of either texts only, or combined with web-based modules.

Half of the studies utilised tailoring or personalisation of the interventions (DeMartini et al., 2018; van der Velde et al., 2021; Mundi et al., 2015; Nolan et al., 2019; Low et al., 2020). In one study (Low et al., 2020), the intervention was completely based on personalised data gathered via a smartwatch, and in another the intervention was dynamic based on patients’ operation date (van der Velde et al., 2021). Two of the text message interventions allowed participants to send messages to the system and receive automated tailored responses (DeMartini et al., 2018; Nolan et al., 2019), and one intervention included tailored ecological momentary assessment messages based on baseline characteristics (Mundi et al., 2015).

#### Theoretical frameworks

Three reports explicitly mentioned that interventions were based on theories for behaviour change (DeMartini et al., 2018; Krebs et al., 2019; McCrabb et al., 2017). The theories mentioned were social cognitive theory (Bandura, 1989), the transtheoretical model (Prochaska, 1979) and the relapse prevention model (Lowman et al., 1996). Other reports mentioned that interventions were based on previous research, clinical best practice, guidelines and expert knowledge (Bendtsen et al., 2019; Nolan et al., 2019). Van der Velde et al. (van der Velde et al., 2021) reported that their app were based on behaviour change techniques (BCTs), for example goalsetting, social support and feedback on behaviour. Also, McCrabb et al. (McCrabb et al., 2017) reported that their intervention employed a number of BCTs, but these were not specified. For additional details of the interventions, please see Additional file 2: Appendix B.

#### Feasibility studies

The six feasibility studies measured subjects’ knowledge, experience, usability, acceptability, satisfaction and perceived helpfulness with respect to the digital interventions (Mundi et al., 2015; McCrabb et al., 2017; Nolan et al., 2019; Low et al., 2020; Kulinski & Smith, 2020; Thomas et al., 2020). All studies utilised study specific questionnaires, resulting in high heterogeneity of measurements. Overall, the feasibility studies reported mixed findings with high user acceptability and satisfaction with the support offered, along with challenges in engaging users. Nolan et al. (Nolan et al., 2019) reported that a majority (82%) of the participants were satisfied with the text messaging smoking cessation program, and that many (68%) expressed that they would be interested in using it again for future surgeries. Similar results were found for the text message intervention by Kulinski et al. (Kulinski & Smith, 2020), who tested a prehabilitation program for patients with obesity awaiting elective surgery. Fifteen of 18 participants (83%) found the programme useful and all participants stated that they would recommend the programme to others in the lead-up to surgery. Mundi et al. (Mundi et al., 2015) found that bariatric surgical participants felt that the intervention helped them understand behaviours of long-term weight loss, and made them feel more connected to the care team. McCrabb et al. (McCrabb et al., 2017) reported that only 11/20 orthopaedic trauma patients remembered using the smoking cessation support, likely due to effects of medication, and very few participants accessed the intervention after hospital discharge, which made evaluation of the intervention problematic. Qualitative data from Low et al. (Low et al., 2020) reported that participants found physical activity prompts to be motivating before abdominal cancer surgery, but also reported that it was especially difficult to walk in the hospital immediately after surgery when they were too weak to walk unassisted, were in the middle of tests or other care procedures, or were on medications that made it difficult to get up and walk. The qualitative study from Thomas et al. (Thomas et al., 2020) showed that elective surgery patients were open and receptive to receive smoking cessation support in a digital format from a health care provider ahead of surgery. Data showed that patients having elective surgery had a strong motivation to quit smoking, where for instance one participant reported: “And then I walked down to her at the unit straight away from meeting with the surgeon to speak with her. And then I said, “Let’s throw away the cigarettes”.

#### Engagement data

Six of the studies (DeMartini et al., 2018; Krebs et al., 2019; Mundi et al., 2015; McCrabb et al., 2017; Nolan et al., 2019; Low et al., 2020) reported on engagement data, defined by Perski et al. (Perski et al., 2017) as “Engagement with DBCIs is (1) the extent (e.g. amount, frequency, duration, depth) of usage and (2) a subjective experience characterised by attention, interest and affect”*.* Among text message interventions where participants were expected to respond to messages (DeMartini et al., 2018; Mundi et al., 2015; Nolan et al., 2019), total response rates ranged from 31 to 81%, with more participants responding early in the intervention period (Nolan et al., 2019). Interventions not using text messages (game and web-based program) (Krebs et al., 2019; McCrabb et al., 2017) showed participation rates of 40–90% with respect to engaging with the intervention at least once. Overall, a majority of studies found a severe drop-off in usage of the interventions after surgery, for instance Low et al. (Low et al., 2020) reported a significant decline from 91% before surgery to 36% during inpatient time to 65% post-discharge.

#### Behaviour change outcomes

The RCT studies (Lemanu et al., 2018; DeMartini et al., 2018; Krebs et al., 2019; van der Velde et al., 2021) examined the effects on behaviour change with respect to physical activity, alcohol and smoking. Lemanu et al. (Lemanu et al., 2018) measured adherence to exercise advice (30 min of light to moderate exercise a day, 5 days a week) via the International Physical Activity Questionnaire (IPAQ) among patients on waiting list for laparoscopic sleeve gastrectomy. Result showed that adherence to exercise advice was significantly higher in exposure group than control group (*p* = 0.041), but there was no evidence suggesting a difference between the two groups at 6 weeks post-operative follow-up. DeMartini et al. (DeMartini et al., 2018) objectively measured alcohol consumption (e.g., ethyl glucuronide) at 8 weeks among pre-liver transplant candidates with alcoholic liver disease. Result showed that none of the individuals tested positive for alcohol consumption at 8 weeks in the intervention group; however, 2 out of 6 participants (33%) in the standard care group did test positive. Krebs et al. (Krebs et al., 2019) measured smoking abstinence (biochemically verified) at 1 month following hospital discharge among cancer patients undergoing surgery. Confirmed smoking abstinence was higher in the intervention group (4/13) in relation to control group (2/11) at 1 month-follow up. Finally, van der Velde et al. (van der Velde et al., 2021) measured change in health risk behaviours (physical activity, alcohol, and smoking) 3 days prior to surgery, based on the recommendations of the Dutch Health Council (questionnaire not disclosed). Compared with the control group, the intervention group showed an increase in self-reported physical activity and muscle strengthening activities prior to surgery. Also, 2 of 2 frequent alcohol users in the intervention group versus 1 of 9 in the control group drank less alcohol prior to surgery. No difference was found in change of smoking cessation.

Difficulties of recruitment were apparent across studies, and sample sizes were relatively small, (*n* = 102) for the full-scale RCT (Lemanu et al., 2018) and (*n* = 15–86) for the pilot-studies (DeMartini et al., 2018; Krebs et al., 2019; van der Velde et al., 2021). Krebs et al. (Krebs et al., 2019) reported not meeting sufficient number of eligible patients despite screening for 2 years.

#### Protocols

Bendtsen et al., 2019. (Bendtsen et al., 2019) reports a protocol for an ongoing RCT of a text message based smoking cessation intervention for patients prior to elective surgery. A total of 434 participants are planned to be randomised, and outcomes are measured subjectively via questionnaires.

## Discussion

### Summary of evidence

This scoping review aimed to identify existing research done in the area of perioperative DBCIs, in particular with respect to alcohol, diet, physical activity and smoking. We identified a limited number of studies (11 studies) across five different surgery contexts (including unspecified elective surgery). Most interventions targeted smoking cessation, were administered preoperatively, and were delivered via text messages. Overall, the findings of this scoping review indicate a paucity of research on the effects of perioperative digital interventions for lifestyle behaviour change. Also, small sample sizes and the use of a variety of outcome measures prevent formal synthesis of results in for instance meta-analyses and make generalisation of findings difficult.

In a wider context, patients’ have been found to have a substantial desire to modify behaviours for postoperative benefit (McDonald et al., 2019), but also a need for structured preoperative support. In addition, patients have been found to have positive expectations of e-Health in preoperative care (van der Meij et al., 2017). However, despite patient acceptance and willingness, small sample sizes due to difficulties of study recruitment prior to surgery causes issues with validity of findings. Thus, there seems to be a discrepancy between patient expectations and acceptance of study procedures. Most studies included in this scoping review had small sample sizes, and outcome and engagement findings should therefore be viewed with high scepticism. Also, some studies have relied on pre-post measurements (Mundi et al., 2015; Nolan et al., 2019; Kulinski & Smith, 2020) to measure behaviour change, a practice that is vulnerable to regression to the mean and other confounding (Vickers & Altman, 2001).

Another issue identified is the timing of intervention delivery, and adherence to the intervention postsurgery. McCrabb et al. (McCrabb et al., 2017) reported that due to effects from medication, only half of the participants remembered using the intervention in hospital after surgery, and very few participants accessed the intervention after hospital discharge. Also, a majority of the studies included in this review found a severe drop-off in usage of the interventions after surgery, potentially indicating a loss of motivation once the main reason for their behaviour change was removed. This is unfortunate since a healthy lifestyle can improve postoperative recovery **(**Li et al., 2013; Minnella et al., 2016; Mayo et al., 2011; van Rooijen et al., 2019a; van Rooijen et al., 2019b**)**, and reduces the risk of future health issues (World Health Organization, 2018). It is also possible that the reason for surgery motivates behaviour change differently, e.g. obesity surgery is more tightly coupled with change in diet and physical activity in comparison to smoking and orthopaedic surgery. However, findings of this scoping review cannot support such conclusions, thus we leave this for future research to investigate.

Use of e-health in perioperative care, including replacement of face-to-face consultations with telerehabilitation and telemonitoring, has shown promise (van der Meij et al., 2016). Thus, digitalisation has made progress in the surgical context, and research on DBCIs as part of perioperative care should learn from these successes. Perhaps a blended care model where DBCIs become a part of the current treatment is a future model. Although there are indications to assume that the preoperative period might be *a window of opportunity* to change behaviour, one must bear in mind that the preoperative period is a stressful and sometimes painful period for many patients. However, it has been argued (Robinson et al., 2020) that if a surgical teachable moment is exploited correctly, the situation can trigger sustainable behaviour change – just as the decision to undergo surgery can be life-changing itself.

### Limitations

We took a broad approach in this review, aiming to identify existing research in the field rather than addressing any specific question. It should be noted that we only searched for reports published in English in peer-reviewed journals, which means that grey literature, conference proceedings and abstracts have not been considered for inclusion. Due to this limitation, there may for instance be local government reports written in other languages than English which are not included in this scoping review.

Overall, the included studies had relatively few participants and suffered from attrition and loss of engagement, a main finding of this scoping review, thus the overall findings with respect to satisfaction and usability should be carefully considered in light of this. Also, use of study specific questionnaires and pre-post measurements questions both the internal and external validity of findings.

## Conclusions

This scoping review highlights the relatively new research field of DBCIs for perioperative behaviour change. Included studies indicate participant satisfaction, but also show recruitment and timing-delivery issues, as well as low retention to the intervention post-surgery. Small sample sizes and the use of a variety of feasibility outcome measures hinders the synthesis of results and makes generalisation difficult.

## Supplementary Information

**Additional file 1: Appendix A.** Search Strategies

**Additional file 2.** Appendix B. TIDieR checklist.

## Data Availability

Not applicable.

## Abbreviations

RCT: Randomised controlled trial
BCT: Behaviour change techniques
PICOS: Population, intervention, comparator, outcomes and study design
DBCI: Digital behaviour change interventions
IPAQ: International Physical Activity Questionnaire
TIDieR: Template for Intervention Description and Replication
