# Supplementary material for: Perioperative digital behaviour change interventions for reducing alcohol consumption, improving dietary intake, increasing physical activity and smoking cessation: a scoping review
Source: Perioper Med (Lond). 2021 Jul 6;10:18. doi: 10.1186/s13741-021-00189-1 (PMC8258960; doi:10.1186/s13741-021-00189-1)
Supplement: Supplementary file 1 — Additional file 1: Appendix A. Search Strategies [file 13741_2021_189_MOESM1_ESM.docx]

# Appendix A – Search strategies

This appendix contains the search strategy used for PubMed, which was searched on September 25 of 2019, and repeated on February 17, 2021. This strategy was adapted to the other information sources (see Methods).

## PubMed

#1 text messaging[tiab] OR text message[tiab] OR text messages[tiab] OR text message-based[tiab]

#2 sms[tiab] OR short message service[tiab] OR short messaging[tiab] OR short message[tiab] OR short messages[tiab]

#3 app[tiab] OR apps[tiab]

#4 cell phone[tiab] OR smartphone[tiab] OR mobile[tiab] OR mobile phone[tiab] OR mobile phone-based[tiab]

#5 ecological momentary assessment[tiab]

#6 telemedicine[tiab] OR ehealth[tiab] OR e-health[tiab] OR electronic health[tiab] OR mhealth[tiab] OR m-health[tiab] OR mobile health[tiab]

#7 digital intervention[tiab] OR digital interventions[tiab]

#8 text messaging[mesh][tiab] OR telemedicine[mesh][tiab] OR ecological momentary assessment[mesh][tiab] OR smartphone[mesh][tiab] OR mobile applications[mesh][tiab] OR cell phone[mesh][tiab]

#9 (#1 OR #2 OR #3 OR #4 OR #5 OR #6 OR #7 OR #8)

#10 health behavior[tiab] OR health behaviors[tiab] OR health behaviour[tiab] OR health behaviours[tiab]

#11 risk behavior[tiab] OR risk behaviors[tiab] OR risk behaviour[tiab] OR risk behaviours[tiab]

#12 health risk behavior[tiab] OR health risk behaviors[tiab] OR health risk behaviour[tiab] OR health risk behaviours[tiab]

#13 risk factors[tiab] OR risk reduction[tiab]

#14 life style[tiab] OR lifestyle[tiab]

#15 harm reduction[tiab]

#16 behavior change[tiab] OR behavior changes[tiab] OR behaviour change[tiab] OR behaviour changes[tiab]

#17 diet[tiab] OR dietary[tiab] OR nutrition[tiab] OR nutrient[tiab] OR nutritious[tiab]

#18 exercise[tiab] OR physical fitness[tiab] OR sedentary[tiab] OR physical activity[tiab] OR physical activities[tiab] OR physically active[tiab] OR physically inactive[tiab] OR physical inactivity[tiab]

#19 smoking[tiab] OR tobacco[tiab] OR cigarette[tiab] OR cessation[tiab]

#20 alcohol consumption[tiab] OR weekly consumption[tiab] OR binge drinking[tiab] OR heavy episodic drinking[tiab]

#21 risky drinking[tiab] OR risky drinkers[tiab] OR risky alcohol consumption[tiab] OR harmful drinking[tiab] OR harmful drinkers[tiab] OR hazardous drinking[tiab] OR hazardous drinkers[tiab]

#22 (#10 OR #11 OR #12 OR #13 OR #14 OR #15 OR #16 OR #17 OR #18 OR #19 OR #20 OR #21)

#23 surgery[tiab] OR surgical[tiab] OR elective[tiab] OR operation[tiab]

#24 operative[tiab] OR preoperative[tiab] OR pre-operative[tiab] OR perioperative[tiab] OR peri-operative[tiab] OR postoperative[tiab] OR post-operative[tiab]

#25 surgical procedures, operative[mesh][tiab] OR postoperative period[mesh][tiab] OR elective surgical procedures[mesh][tiab] OR general sugery[mesh][tiab] OR surgery[mesh][tiab]

#26 (#23 OR #24 OR #25)

#27 (#9 AND #22 AND #26)
